# The Use of Passive Straight Leg Raising Test: A Survey of Clinicians

**DOI:** 10.5704/MOJ.1511.012

**Published:** 2015-11

**Authors:** K Pande

**Affiliations:** Clinical Specialist in Orthopaedics, Raja Isteri Pengiran Anak Saleha Hospital, Negara Brunei Darussalam

**Keywords:** Survey, Passive Straight Leg Raising Test, Low back pain, Radiculopathy, Diagnosis

## Abstract

Introduction: Passive Straight Leg Raising Test (PSLRT) is one of the most commonly performed test in clinical practice. The purpose of this study was to survey the practice and interpretation of PSLRT amongst clinicians working in a tertiary care hospital.

Methods: A 15 item questionnaire survey was developed covering various aspects of PSLRT. Orthopaedic surgeons(n=15), neurosurgeons (n=7) and physiotherapists (n=9)were identified as clinicians performing this test regularly and were approached to take part in the survey.

Results: The PSLRT was used in all cases of back and leg pain by 68% and correctly performed by 30/31. There was a wide variation in the angle at which it was considered positive (median 45 degrees; range 10-90 degrees). Only 7/31 correctly recognised reproduction of leg pain as indicative of a positive PSLRT. The sitting /distraction SLRT andwell leg / cross SLRT was performed only by 3/31 and 16/31 of clinicians respectively. 90% felt that a positive PSLRT suggested nerve root irritation and 57% thought it was due to stretch of dura and / or nerve root. 23/31 clinicians felt that PSLRT was useful or very useful and 90% reported that result of PSLRT would affect the way they treat a patient.

Conclusions: PSLRT is widely used, correctly performed and felt to be useful in practice. But the interpretation of a positive test, understanding of its mechanism and useof variations is poor. There is a need to improve the interpretation and understanding of PSLRT amongst its users.

## Introduction

Low back pain is a common reason for consulting a clinician and across the world it is a major cause for absence from work^1^. Between 1.2 to 44% of patients have ‘Sciatica’ or radiating pain in the lower limb, which may result from a herniated lumbar disc^2^.

The clinical evaluation of a patient with radicular pain / sciatica includes adequate history and clinical examination. Passive Straight Leg Raising Test (PSLRT) is one of the most common tests used in clinical practice^3-5^. However together with other clinical signs used in practice for evaluation of a patient with lumbar radiculopathy, it was found to be of limited utility when used in isolation^4^, and with low reproducibility when used in general practice^6^. Rebain *et al* following a systematic review concluded that ‘there remains no standard PSLRT procedure, no consensus on interpretation of results and little recognition that a negative PSLRT outcome may be of greater diagnostic value than a positive one’^7^.

For a clinical test subject to individual interpretation, only two surveys, one each in osteopaths and chiropractors has reported their perceptions and understanding of the test^5,8^. No data is available on perception and awareness in other clinicians who regularly use PSLRT in practice.

The aim of the present study was to survey the practice and interpretation of PSLRT amongst orthopaedic surgeons, neurosurgeons and physiotherapists working in a tertiary care hospital.

## Materials and Methods

Based on review of relevant literature, a questionnaire was developed with 15 items. These covered the areas of performing and interpretation of a positive PSLRT including site and angle of pain reproduction, understanding of mechanism of PSLRT, use of variations of straight leg raising test (SLRT) in practice and understanding of the significance of a positive and negative PSLRT (Appendix A). The questionnaire was piloted amongst 5 surgeons not taking part in the survey for comprehension and appropriate changes were made to the text.

Orthopaedic surgeons, neurosurgeons and physiotherapists working in a tertiary care hospital in Negara Brunei Darussalam were identified as practitioners who routinely use PSLRT in the assessment of patients with low back pain and radiculopathy. The questionnaire was anonymous and each practitioner was approached individually to explain the purpose of the survey, obtain verbal consent and requested to complete the questionnaire.

The sample covered all the clinicians working in the respective departments at the time of the survey. As the number of potential participants was small, it was decided to approach them individually. Participation was voluntary and none of the clinicians approached declined to participate.

To assess the effect of experience on the interpretation of PSLRT and use of its variations, the sample was divided into clinicians with less than 10 year experience (n=17) and with more than 10 year experience (n=14).

The data was entered in excel chart and analysis done using SPSS version 10.0. The results are presented as descriptive results.

## Results

The sample consisted of orthopaedic surgeons (n= 15), neurosurgeons (n=7) and physiotherapists (n= 9).

It was noted that 30/36 performed the PSLRT as per the recommended procedure but there was a wide variation in the angle at which it was deemed positive (median 45 degrees; range: 10-90 degrees). Most of those surveyed (30/31) performed PSLRT with ankle dorsiflexion. Only 3/31 performed the sitting / distraction SLRT while 23/31 did not and 5/31 were not aware of this test. Only about 50% of sample performed the well / cross leg SLRT.

Only 7/31 recognised reproduction of leg pain as indicative of a positive PSLRT. 18/31 felt that reproduction of both back and leg pain and 6/31 felt production of back or buttock pain was important for a positive test.

Majority (28/31) of those surveyed felt that a negative PSLRT does not rule out an intervertebral disc prolapse while 2/31 felt that a negative PSLRT rules it out. A total 34.5% of those surveyed felt that PSLRT varies with age while equal number felt that age has no effect on it.

**Fig. 1:**
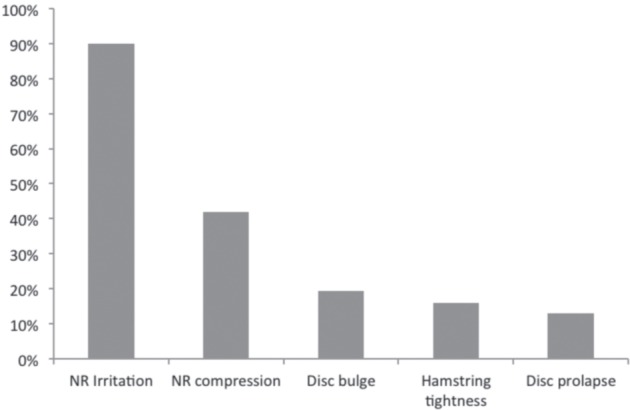
Responses to question about ‘what a positive PSLRT suggested’ (NR: nerve root).

**Fig. 2:**
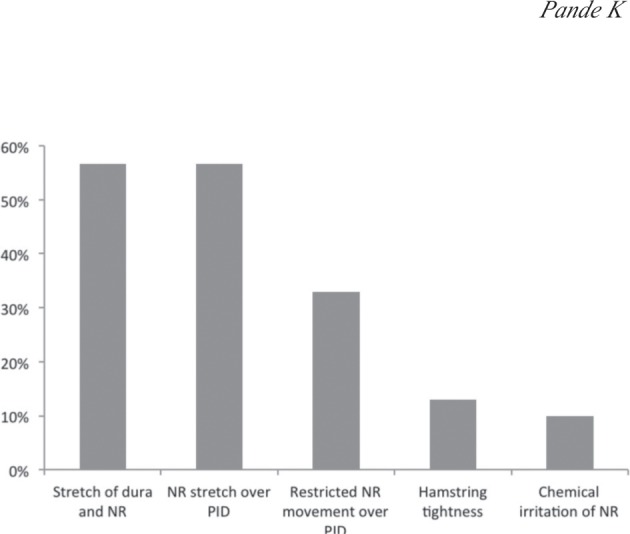
Responses to question about ‘pain producing mechanism of PSLRT’. (NR: nerve root, PID: prolapsed intervertebral disc)

Multiple responses were allowed for the two questions, about ‘what a positive PSLRT suggested’ and ‘pain producing mechanism in PSLRT’. These are presented in Fig. 1 and Fig. 2 respectively as percentage total of responses. Nerve root irritation was reported as the most common interpretation of a positive PSLRT while stretching of the nerve root alone or in combination with the dura was thought as the most common pain producing mechanism. A single pain producing mechanism was chosen by 68% of those surveyed.

**Fig. 3:**
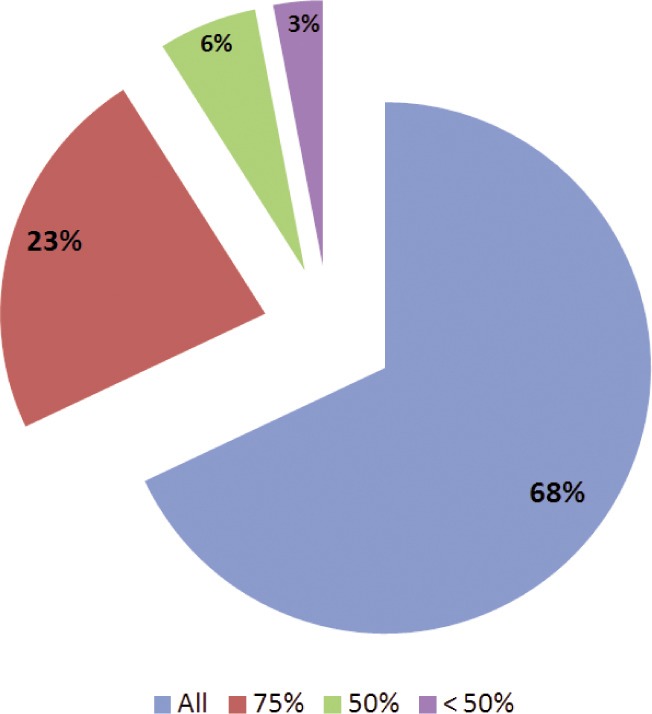
Percentage use of PSLRT in assessment of number of patients.

The percentage use of PSLRT in assessment of patients in practice is given in Fig. 3. Majority of those surveyed (90%) reported that result of PSLRT would affect the way they treat a patient. 13/31 felt that it was a useful clinical test while 10/31 felt it was very useful. One respondent felt that it was of no clinical use.

The number of clinicians correctly recognizing a positive PSLRT as reproduction of leg pain was 4 and 3 respectively in clinicians with less and more experience. There was no difference in the mean ± SD of angle at which PSLRT was considered positive between the two groups (45.4 ± 19.3 degrees vs 41.1 ± 18.6 degrees).

Most clinicians (16/17 and 14/14) reported performing SLRT using dorsiflexion of the foot. None and 5 less experienced clinicians reported using sitting / distraction SLRT and well leg / cross SLRT while 3 and 11 more experienced clinicians reported using these variations respectively.

## Discussion

The present study revealed that amongst the clinicians surveyed, PSLRT was widely used, performed correctly and seen as a useful clinical test impacting on the way a patient is treated. Majority of the clinicians correctly identified that PSLRT suggests nerve root irritation but there was a lack of understanding of its mechanism and wide variation in interpretation of a positive PSLRT both in terms of the angle and reproduction of symptoms. Though large number of clinicians performed PSLRT using ankle dorsiflexion, use of other variations like sitting / distraction SLRT or well / cross leg SLRT was very low. Experience of clinician did not impact on the interpretation of PSLRT but with experience more clinicians were using variations of SLRT.

The historical aspects of PSLRT and its variations have been extensively reviewed^7,9^. These authors have also reviewed the mechanism of pain production during SLRT. While different methods of performing PSLRT have been reported, the protocol advocated by Breig and Troup is most widely accepted^7^.

### Performance characteristics of PSLRT

PSLRT has high sensitivity and but low specificity in patients undergoing surgery for disc prolapse^4^. In these patients the prevalence of disc herniation is also high and therefore this observation cannot be applied to other patient population. In a general practice setting the reproducibility of SLRT was found to be low^6^. Even in specialized care setting, Iverson et al have reported low accuracy of various clinical tests including SLRT in patients with chronic lumbar radiculopathy^10^. It is now recognized that the prevalence of a disease can lead to variation of a tests sensitivity and specificity^11^.

A systematic review concluded that the straight leg raising test has low specificity, therefore limiting its diagnostic accuracy. The pooled sensitivity of the SLRT was 91% with a pooled specificity of 26%^12^. A more recent systematic review to assess clinical utility of SLRT has shown a wide variation in the sensitivity and specificity of the test partly due to differing reference standards used. It was also noted that hamstring tightness can give rise to a falsely high sensitivity of the SLRT^13^.

### Results of similar survey available in literature

A survey of Osteopaths revealed that SLRT was commonly used and correctly performed in their practice. While a positive SLRT was identified as reproduction of pain in the affected limb, there was wide variation in the interpretation of angle. There was poor agreement on the mechanism of SLRT and its diagnostic implications^8^.

In another survey to examine the perceptions of clinical value of five commonly used orthopaedic tests including PSLRT amongst faculty of a chiropractic college, faculty members significantly agreed that a positive SLRT was a strong indicator of presence of disc pathology. Only 32% of faculty agreed than a negative SLRT suggests absence of disc pathology^5^.

### Performing and Interpreting PSLRT

Consistent with the literature this study revealed that PSLRT is widely used but there was wide variation in its interpretation^7,8^. A positive SLRT is reproduction of the radicular pain at an angle between 35 to 75 degrees, when the majority of movement of the nerve occurs at the intervertebral foramen^9^. Clinicians surveyed reported a range from 10 to 90 degrees for interpretation of a positive test with only 7/31 correctly identifying reproduction of leg pain as a positive test.

The interpretation of angle for a positive SLRT should be seen in the context of study by Boyd and Villa, who have noted that the normal variation in the straight leg raising range is high, between 40 to 85 degrees^14^. Thus for interpretation of a positive PSLRT, site of pain is also significant.

The sitting / distraction SLRT has been shown to be more sensitive than PSLRT^15^ with another study reporting substantial agreement and good correlation between the two^16^. In contrast Rabin *et al* found that the traditional SLRT was better^17^. Deville *et al* found that cross SLRT has higher specificity of 88% but a very low sensitivity of only 29%^12^. Thus the role of performing various modifications of SLRT is well established but its usage in the present sample was low.

A review by Rebain *et al* and Capra *et al* have shown that the discriminative power of SLRT decreases with increasing age^7,18^. This fact was correctly identified by only about 1/3 rd of the clinicians surveyed.

Rebain *et al*^7^ have concluded based on review of various studies that a negative PSLRT may be of greater diagnostic value than a positive one but the present study revealed that this was not clearly understood by the clinicians surveyed. This is also evident from a systematic review^7^ and survey of osteopaths^8^.

### Mechanism of PSLRT

The various mechanisms of limitation of PSLRT have been summarized by Urban^9^ and Rebain *et al*^8^. Pain production in a positive PSLRT has been attributed to compression of the nerve root, inflammation of the dural cuff of the nerve root, intervertebral venous congestion and defensive hamstring muscle tightness.

While a positive SLRT is indicative of nerve root tension or mechanosensitivity^14^, various studies in literature have correlated it with the diagnosis of lumbar disc prolapse seen either on magnetic resonance imaging (MRI) or intraoperatively.

It is well recognized that disc herniation may be seen in asymptomatic subjects on MRI^19^. Vroomen *et al*^3^ and Iverson *et al*^10^ have shown that a positive SLRT is not predictive of nerve root compression seen on MRI while Capra *et al*^18^ have shown low accuracy of the SLRT in diagnosing disk herniation when compared with results of MRI. Similarly Li and Yen have shown a poor association between clinical diagnosis of disc herniation and radiologic abnormalities noted on MRI^20^.

In the present study, clinicians were aware that a positive PSLRT suggests nerve root irritation though other interpretations were also cited. Their understanding of mechanism of PSLRT was poor.

### Usefulness of PSLRT in practice

The results of the present survey are consistent with the survey of osteopaths, a large number of whom routinely used SLRT and based their treatment decision on it. Experience of the clinician was not seen to affect the interpretation of a positive PSLRT. Variation of SLRT with dorsiflexion of foot was commonly used while experienced clinicians were more likely to use the other variations.

The findings of the present survey and information in the literature question the use of PSLRT by clinicians. It is widely used but subject to varied interpretations. The understanding of its mechanism is less than satisfactory and it has been found to have variable sensitivity and specificity. There is clearly a need for research into the clinical use of SLRT in terms of definition, interpretation and understanding of mechanism as recommended by Rebain *et al*^7^.

A limitation of the study was a small sample size dictated by the number of clinicians working in the hospital. The findings of this study need to be confirmed in a larger sample. The questionnaire did not explore the opinion about use of PSLRT in assessing postoperative outcome and need for reoperation.

## Conclusion

PSLRT is widely used, performed correctly and seen as a useful clinical test impacting on the way a patient is treated amongst the clinicians surveyed. Clinicians are aware of what a positive test suggests but there is a lack of understanding of its mechanism, wide variation in interpretation of a positive PSLRT and poor use of variations of SLRT. With experience, clinicians use the variations of SLRT more but it has no impact on the interpretation of PSLRT.
